# Artery-first vs vein-first surgical technique for segmentectomy of non-small cell lung cancer: A protocol for systematic review and meta-analysis

**DOI:** 10.1097/MD.0000000000022206

**Published:** 2020-10-09

**Authors:** Zhangwei Tong, Jiekun Qian, Xiaojie Yang, Lin Jiangbo

**Affiliations:** Department of Thoracic Surgery, Fujian Medical University Union Hospital, Fuzhou, China.

**Keywords:** artery-first surgical technique, non–small cell lung cancer, segmentectomy, Vein-first surgical technique

## Abstract

**Background:**

Surgery for lung cancer squeezes the tumor, further promoting the circulation of tumor cells, which may be one of the reasons for lung cancer metastasis and recurrence. In theory, the potential risk of tumor cell proliferation can be minimized if the outflow veins are ligated first (via veins first [V-first]) rather than arteries first (via arteries first [A-first]). However, due to the lack of sufficient evidence, this technical concept has not been widely accepted as a standard in surgical oncology in the current guidelines. This systematic review and meta-analysis will be used to determine which techniques will yield longer patient survival and benefit patients during segmentectomy.

**Methods:**

We will search PubMed, Web of Science, Embase, Cancerlit, the Cochrane Central Register of Controlled Trials, and Google Scholar databases for relevant clinical trials published in any language before January 1, 2021. Randomized controlled trials (RCTs), quasi-RCTs, propensity score-matched comparative studies, and prospective cohort studies of interest, published or unpublished, that meet the inclusion criteria will be included. Subgroup analysis of the type of operation, tumor pathological stage, and ethnicity will be performed.

**Results:**

The results of this study will be published in a peer-reviewed journal.

**Conclusion:**

As far as we know, this study will be the first meta-analysis to compare the efficacy of the vein-first and artery-first surgical technique of segmentectomy for patients diagnosed with resectable non–small cell lung cancer. Due to the nature of the disease and intervention methods, RCTs may be inadequate, and we will carefully consider inclusion in high-quality, non-RCTs, but this may result in high heterogeneity and affect the reliability of the results.

INPLASY registration number: INPLASY202080062

## 1 Introduction

Surgery is the first choice for many solid tumors, such as non-small cell lung cancer (NSCLC), esophageal cancer, liver cancer and so on. However, even after radical resection, about 50% of patients will develop local recurrence or distant metastasis within 3 years.^[1–4]^

Many studies have shown that surgical procedures can promote the spread of tumor cells into the circulatory system.^[5–9]^ In the process of lung cancer surgery, the tumor can be squeezed and further promote the tumor cells to the circulation, which may be one of the reasons for lung cancer metastasis and recurrence. McCulloch et al reported that tumor cells could be detected in venous blood during operation.^[10]^ In addition, lung cancer is also common tumor vascular invasion, which may be the reason for the high incidence of hematogenous dissemination of tumor cells.^[11–13]^ One surgical technique to prevent tumor cells from spreading into the bloodstream is to ligate the outflow vein.^[14]^

In theory, the potential risk of tumor cell proliferation can be minimized if the outflow vein is ligated first (via v-first) rather than the artery (via a-first). However, due to the lack of sufficient evidence, this technical concept has not been widely accepted as a standard in surgical oncology in the current guidelines. This systematic review and meta-analysis will be used to determine which techniques can prolong the survival time of patients undergoing segmentectomy and are more beneficial to patients with resectable NSCLC.

## 2 Objective

We will conduct a systematic review and meta-analysis to assess the efficacy and safety of venous advance and arterial surgical techniques in segmentectomy for patients with resectable NSCLC.

## 3 Methods

This protocol adheres to the Preferred Reporting Items for Systematic Review and Meta-Analysis Protocols statement.^[15]^ The results of this systematic review and meta-analysis will be published with reference to the Preferred Reporting Items for Systematic Review and Meta-Analysis guidelines.^[15]^

## 4 Patient and public involvement

This study will be based on published or unpublished studies, and records and will not involve patients or the public directly.

### 4.1 Eligibility criteria

#### 4.1.1 Types of studies

Randomized controlled trials (RCTs), quasi-RCTs, propensity score matched comparative studies and prospective cohort studies of interest, published or unpublished, will be included. These should be completed, and the efficacy and safety of the vein-first versus artery-first surgical technique of segmentectomy for patients diagnosed with resectable non-small cell lung cancer.

#### 4.1.2 Types of participants

The participants will be patients diagnosed with resectable, pathologically confirmed non-small cell lung cancer who were treated with segmentectomy, and there will be no restrictions on sex, ethnicity, economic status, or education.

#### 4.1.3 Types of interventions

All types of vein-first or artery-first surgical technique of segmentectomy for patients diagnosed with resectable non-small cell lung cancer will be studied.

#### 4.1.4 Types of outcome measures

##### 4.1.4.1 Primary outcomes

The primary outcome will be overall survival of patients with respectable non-small cell lung cancer after surgery.

##### 4.1.4.2 Secondary outcomes

We will evaluate the 5-year survival, recurrence-free survival, and median survival rates as well as the quality of life and complication rate of patients with resectable non–small cell lung cancer after segmentectomy.

### 4.2 Information sources

Two reviewers (ZWT and JKQ) will search PubMed, Web of Science, Cancerlit, Embase, Cochrane Central Register of Controlled Trials, and Google Scholar databases for relevant trials published before October 1, 2020, without any language restrictions.

### 4.3 Search strategy

The subject terms and keywords corresponding to Medical Subject Heading terms will be used to search for eligible trials in the databases as mentioned above with no language restrictions. Search strategies in PubMed are shown in Table 1.

**Table 1:**
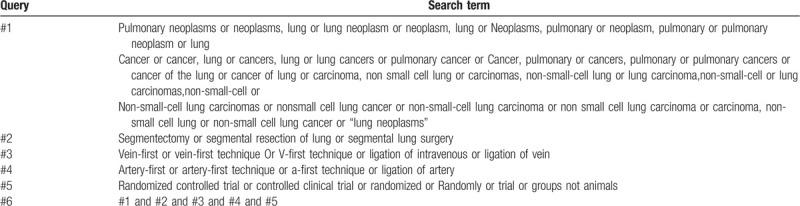
PubMed search strategies.

### 4.4 Data collection and analysis

We will adopt the methods described in the Cochrane Handbook for Systematic Reviews of Interventions to pool the evidence.^[16]^

#### 4.4.1 Study selection

Two authors (ZWT and JKQ) will independently screen each title and abstract of all the papers searched, and the trials that do not meet the inclusion criteria described in this protocol will be excluded. The full text of all the possibly eligible trials will be screened independently and in duplicate by the 2 authors. Trials that are irrelevant or do not meet the inclusion criteria will be excluded. Trials that meet the inclusion criteria and excluded studies along with the reasons for their exclusion will be documented by the 2 authors (ZWT and JKQ). If there is a disagreement between the 2 authors, we will come to a resolution by discussing it with the third author (JBL). If necessary, we will consult the fourth author (JBL) to resolve the disagreement. The selection process will be shown in a Preferred Reporting Items for Systematic Reviews and Meta-Analyses flow chart in detail.

#### 4.4.2 Data extraction and management

We will extract the following data from the included trials.

- Study characteristics: author, publication date, country, study design, randomization, periods of data collection, follow-up duration, withdrawals, and overall duration of study.
- Population characteristics: age, sex, pathology diagnosis, tumor stage, pathologic tumor size, performance status, ethnicity, history of smoking, and inclusion criteria.
- Interventions: type of operation, number of lymph nodes retrieved, extent of resection, duration of operation, bleeding, and postoperative adjuvant therapy.
- Outcomes: overall survival, 5-year survival, recurrence-free survival, median survival, length of stay, length of intensive care unit stay, quality of life, complications, and adverse events.

We will use the pre-designed table to record the data extracted from the included trials. If relevant data from the trials are lost or unclear, we will consult the author via email before determining whether the study is to be included.

### 4.5 Assessment of risk of bias

The Cochrane Handbook for Systematic Reviews of Interventions will be used to assess the risk of bias of each trial included. The 2 authors (ZWT and JKQ) will evaluate the risk of bias based on the following domains: random sequence generation (selection bias), allocation concealment (selection bias), blinding of participants, and personnel (performance bias), blinding of outcome assessment (detection bias), incomplete outcome data (attrition bias), selective outcome reporting (reporting bias), and other biases.^[17]^ The risk of bias in each domain will be assessed as high, low, or uncertain, and the results of the evaluation will be shown on the risk of bias graph. EPOC guidelines will be used to assess the risks of the non-RCTs included.^[18]^

### 4.6 Data analysis

We will use Review Manager and Stata software to synthesize the data extracted. If the data extracted from the included studies are evaluated as highly homogeneous, we will use them to conduct a meta-analysis for the purpose of obtaining a clinically meaningful result. To carry out a standard meta-analysis, we will use the Chi-squared and I^2^ statistical tests to evaluate statistical heterogeneity among the studies. If there is high heterogeneity (*P* < .1 or I^2^ statistic > 50%), we will use the DerSimonian and Laird random effect model to analyze the extracted data. Because high heterogeneity may be caused by different types of tumors and different stages of tumors diagnosed by pathology and different means of adjuvant therapy may be used after the operation, we will perform a subgroup analysis of the types of tumors (esophageal squamous cell carcinoma and esophageal adenocarcinoma), the pathological stages of the tumors, and the means of adjuvant therapy after the operation (types of chemotherapeutic drugs and whether or not radiotherapy is accepted). Otherwise, we will adopt a fixed-effect model to analyze the data. We will adopt the Mantel-Haenszel method to pool the binary data, and the results will be reported in the form of relative risk with a 95% confidence interval. An inverse variance analysis method will be used to pool the continuous data, and the results will be reported in the form of a standardized mean difference with a 95% confidence interval.

#### 4.6.1 Subgroup analysis

If there is substantial heterogeneity and if the available data are sufficient, we will perform subgroup analysis to search for potential origins of heterogeneity. If the extracted data are enough, we will conduct subgroup analysis of the type of operation, type of tumor, tumor stage, age, and postoperative adjuvant treatment.

#### 4.6.2 Sensitivity analysis

We will conduct a sensitivity analysis to evaluate the robustness and reliability of the aggregation results by eliminating trials with a high bias risk. If a reporting bias exists, we will use the methods of fill and trim to analyze for publication bias.^[19]^

### 4.7 Publication bias

Funnel charts and Egger test will be adopted to assess for publication bias if there are no less than 10 eligible trials. If reporting bias is suspected in a trial, we will contact the corresponding author via email to determine whether there are additional outcome data that were not reported.

### 4.8 Evidence evaluation

We will classify the quality of all the evidence into four levels (high, medium, low, and very low) in accordance with the criteria of (study limitations, imprecision, publication bias, indirectness bias, and effect consistency).^[20]^

## 5 Discussion

V-first technology has been proved to reduce the circulation of tumor cells during surgery, and is beneficial to the survival of patients with NSCLC.^[21]^ These better results may be due to a reduction in the number of tumor lobes and the avoidance of squeezing tumor cells into circulation during surgery. In V - first operation, the pulmonary vein at the shallowest part of pulmonary hilum was dissected, and then the branches of bronchus and pulmonary artery were dissected. The operation avoids repeated extrusion and tumor leaf turnover. In addition, once the drainage vein is blocked, tumor cells are less likely to enter the blood.^[22,23]^

Some surgeons have proposed v-priority pneumonectomy. However, some surgeons still believe that the continuous flow of the pulmonary artery into the lobes of the ligated vein leads to a loss of intravascular volume. On the other hand, A-first may have the advantage of preventing unnecessary blood loss from resected lobes.^[24,25]^ If ligating the outflow vein first, rather than ligating the artery first, theoretically, the potential risk of tumor cell proliferation can be reduced to a minimum. However, due to the lack of sufficient evidence, this technical concept has not been widely accepted as a standard in surgical oncology in the current guidelines. This systematic review and meta-analysis will be used to determine which technique in segmentectomy prolongs the survival time and is more beneficial for patients with resectable (NSCLC).

As far as we know, this study will first systematically review and meta-analysis to compare the effectiveness and outcomes of these two different surgical techniques to determine which is more likely to benefit NSCLC patients and clinicians to provide a basis for formulating optimal treatment strategies for patients.

## Author contributions

Jiangbo Lin the guarantor of the article. Zhangwei Tong and Jiekun Qian conceived and designed the study. Zhangwei Tong and Jiekun Qian drafted this protocol. Zhangwei Tong, Jiekun Qian and Xiaojie Yang will perform the search, screening, and extraction. Jiangbo Lin have strictly reviewed this protocol and approved of publication.

**Conceptualization:** Zhangwei Tong, Jiekun Qian.

**Data curation:** Zhangwei Tong, Jiekun Qian, Xiaojie Yang.

**Formal analysis:** Zhangwei Tong, Jiekun Qian.

**Investigation:** Zhangwei Tong.

**Methodology:** Zhangwei Tong.

**Project administration:** Zhangwei Tong.

**Resources:** Zhangwei Tong.

**Software:** Zhangwei Tong.

**Supervision:** Zhangwei Tong, Jiangbo Lin.

**Validation:** Jiangbo Lin.

**Writing – original draft:** Zhangwei Tong.

**Writing – review & editing:** Zhangwei Tong.

## Abbreviations

Abbreviations: NSCLC = non-small cell lung cancer, RCTs = randomized controlled trials.
